# Diabetes mellitus and tuberculosis: programmatic management issues

**DOI:** 10.5588/ijtld.15.0069

**Published:** 2015-08

**Authors:** A. D. Harries, A. M. V. Kumar, S. Satyanarayana, Y. Lin, R. Zachariah, K. Lönnroth, A. Kapur

**Affiliations:** * International Union Against Tuberculosis and Lung Disease (The Union), Paris, France; † London School of Hygiene & Tropical Medicine, London, UK; ‡ The Union South-East Asia Regional Office, New Delhi, India; § The Union China Office, Beijing, China; ¶ Medical Department, Operational Research Unit, Médecins Sans Frontières, Brussels Operational Centre, Luxembourg, Luxembourg; # Global TB Programme, World Health Organization, Geneva, Switzerland; ** Department of Public Health Sciences, Karolinska Institutet, Stockholm, Sweden; †† World Diabetes Foundation, Gentofte, Denmark

**Keywords:** DM, TB, DM-TB interaction, bi-directional screening, programmatic challenges

## Abstract

In August 2011, the World Health Organization and the International Union Against Tuberculosis and Lung Disease launched the Collaborative Framework for Care and Control of Tuberculosis (TB) and diabetes mellitus (DM) to guide policy makers and implementers in combatting the epidemics of both diseases. Progress has been made, and includes identifying how best to undertake bidirectional screening for both diseases, how to provide optimal treatment and care for patients with dual disease and the most suitable framework for monitoring and evaluation. Key programmatic challenges include the following: whether screening should be directed at all patients or targeted at those with high-risk characteristics; the most suitable technologies for diagnosing TB and diabetes in routine settings; the best time to screen TB patients for DM; how to provide an integrated, coordinated approach to case management; and finally, how to persuade non-communicable disease programmes to adopt a cohort analysis approach, preferably using electronic medical records, for monitoring and evaluation. The link between DM and TB and the implementation of the collaborative framework for care and control have the potential to stimulate and strengthen the scale-up of non-communicable disease care and prevention programmes, which may help in reducing not only the global burden of DM but also the global burden of TB.

## RESUME

En août 2011, l'Organisation Mondiale de la Santé et l'Union Internationale Contre la Tuberculose et Maladies Respiratoires ont lancé le Cadre Collaboratif pour la Prise en charge de la Tuberculose (TB) et du Diabète (DM) et la lutte contre ces maladies afin de guider les responsables des politiques et les responsables de la mise en œuvre dans leur combat contre ces deux épidémies. Des progrès ontété réalisés et incluent la recherche de la meilleure manière d'entreprendre le dépistage des deux maladiesà la fois, la façon d'offrir un traitement et des soins optimaux aux patients atteints des deux maladies et le cadre le plus adapté pour le suivi et l'évaluation. Les défis programmatiques principaux incluent les questions suivantes : le ciblage du dépistage pour tous les patients ou seulement ceux qui présentent des caractéristiques de risque élevé ; les techniques les plus adaptées au diagnostic de la TB et du DM en contexte de routine ; le meilleur moment de dépister un DM chez les patients ; la manière de fournir une approche intégrée et coordonnée à la prise en charge des cas ; et finalement la façon de persuader les programmes de maladies non transmissibles d'adopter une approche d'analyse de cohorte, en utilisant de préférence des dossiers médicaux électroniques, pour le suivi et l'évaluation. Le lien entre DM et TB et la mise en œuvre du Cadre Collaboratif pour les soins et la lutte ont le potentiel de stimuler et de renforcer l'expansion des programmes de traitement et de prévention des maladies non transmissibles, ce qui pourrait contribuer à réduire non seulement le poids global du DM mais également le fardeau de la TB.

## RESUMEN

En agosto del 2011, la Organización Mundial de la Salud y la Unión Internacional Contra la Tuberculosis y Enfermedades Respiratorias pusieron en marcha un Marco de Colaboración para la Atención y Control de la Tuberculosis (TB) y la Diabetes (DM), con el objeto de orientar a las instancias encargadas de formular las políticas y de ponerlas en práctica, en materia de lucha contra la epidemia de ambas enfermedades. Se han logrado progresos en la determinación de los mejores mecanismos de detección sistemática bidireccional de ambas enfermedades, el suministro óptimo de tratamiento y atención a estos pacientes y en la definición del marco más conveniente de seguimiento y evaluación. Los aspectos programáticos fundamentales que plantean dificultades consisten en decidir si la detección sistemática debe abarcar a todos los pacientes o si debe dirigirse a los que presentan características de alto riesgo; escoger las técnicas diagnósticas más adaptadas al diagnóstico de la TB y la DM en la práctica corriente; definir el momento más oportuno para investigar la DM en los pacientes con diagnóstico de TB; escoger el mejor método para ofrecer un enfoque integrado y coordinado del tratamiento de los casos; y porúltimo, persuadir a los encargados de los programas de enfermedades no transmisibles de que adopten la estrategia del análisis de cohortes, de preferencia a partir de los registros médicos informatizados, con fines de seguimiento y evaluación de los pacientes. El establecimiento del vínculo entre la DM y la TB y la introducción de un marco de trabajo conjunto de atención y control pueden fomentar y reforzar la ampliación de escala de los programas de atención y prevención de las enfermedades no transmisibles y con ello no solo se disminuirá la carga mundial de morbilidad por DM, sino también la carga mundial de la TB.

IN 2007 AND 2008, two systematic reviews of the medical literature alerted the scientific community to the important association between diabetes mellitus (DM) and tuberculosis (TB).^1^,^2^ The studies demonstrated that the relative risk of TB in cohorts of DM patients compared with normal subjects was 3.1 (95% confidence interval 2.3–4.3), and that the odds ratios of TB occurring in persons with DM in case-control studies varied from 1.2 to 7.8. Further reviews have confirmed these findings, and suggest that the overall risk of TB in persons with DM is two to three times higher than in the general population.^3^,^4^ Both type 1 and type 2 DM can increase the risk of TB, but as type 2 disease accounts for ⩾90% of DM cases worldwide, the public health burden of comorbid disease from type 2 DM is much greater, and this is the focus of this paper.^5^

Although the link between the two diseases has been known for years from anecdotal reports, case studies and clinical experience, the implications of this interaction for public health were thought until recently to be insignificant, as TB is relatively rare in high-income countries where DM is prevalent, and DM is perceived as being a minor problem in low-and middle-income countries (LMICs) where TB is epidemic. This perception has changed radically in the last decade with the recognition of the huge, unfolding epidemic of DM in LMICs, the slower decline in global TB incidence than would be expected from epidemiological modelling and a better understanding of how DM and TB interact.

It is not clear why DM patients, particularly those with poorly controlled disease, are at increased risk of TB, although changes have been found in both their innate and their adaptive immune responses.^1^,^6^ The exact mechanisms underlying this susceptibility to TB are still relatively undefined and are in need of detailed evaluation. In 2012, the population attributable fraction of DM among adult TB cases was estimated at 15%, with the number of adult TB cases associated with DM being 1 042 000, only slightly less than observed for human immunodeficiency virus (HIV) associated TB.^7^ The top 10 countries with the highest incidence of TB associated with DM are shown in Table 1.

**Table 1.**
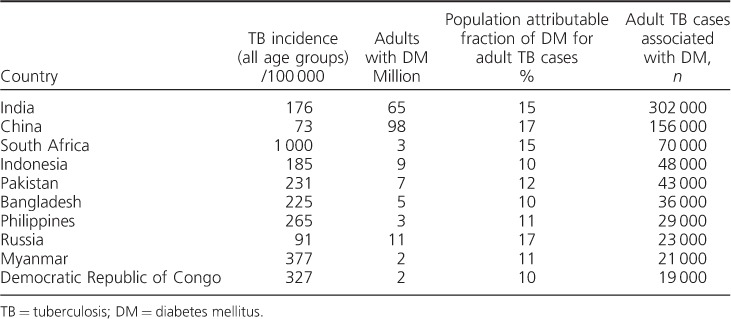
Top 10 countries with the highest incidence of TB associated with DM (adapted from Lönnroth et al.^7^)

In addition to the increased risk for TB, persons with dual disease have worse anti-tuberculosis treatment outcomes with longer times to sputum culture conversion, increased risk of death or treatment failure, and increased risk of recurrent TB after successful completion of treatment.^8^,^9^ Conversely, TB, like other infections, can worsen glycaemic control and complicate the clinical management of DM. Bidirectional screening and integrated management should help to improve early diagnosis, treatment and health outcomes of both conditions. In the light of this situation, the World Health Organization (WHO) and the International Union Against Tuberculosis and Lung Disease (The Union) launched the Collaborative Framework for Care and Control of Tuberculosis and Diabetes in August 2011 to guide policy makers and implementers in combatting the TB-DM epidemic (Table 2), with emphasis on operational (and other) research so that the evidence base for action can be built and strengthened.^10^ This has had the desired effect, with a multitude of studies being conducted in the last few years, as a result of which a number of programmatic issues and challenges have been and are continuing to be identified.

**Table 2.**
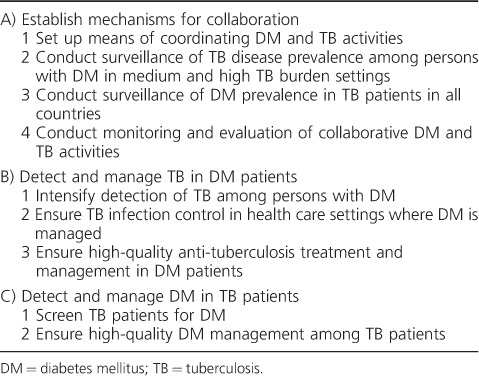
Collaborative activities to reduce the dual burden of DM and TB (adapted from the Collaborative Framework for Care and Control of Tuberculosis and Diabetes^10^)

In the present paper, we highlight these challenges in relation to 1) bi-directional screening of TB and DM, 2) case management, and 3) monitoring and evaluation.

## SCREENING TUBERCULOSIS PATIENTS FOR DIABETES MELLITUS

Where resources for DM diagnosis are available, TB patients should be screened for DM at the start of anti-tuberculosis treatment.^10^ A systematic review of bidirectional screening for DM and TB in 2009 using strict inclusion criteria identified 18 studies on screening TB patients for DM, with a yield of DM that ranged from 1.9% to 35%, suggesting that the value of the activity depends to a large extent on where the screening is taking place geographically.^11^ Since this review, various countries have reported a high yield from screening; these include India (especially southern India),^12–15^ Pakistan,^16^ China,^17^,^18^ Mexico,^19^ the United States,^20^ Tanzania,^21^ Nigeria^22^ and the Republic of the Marshall Islands in the Pacific.^23^ However, as high yields are not always the case,^24^ programmes will need to decide on whether such screening is needed and, if so, what is the most cost-effective approach and whether targeted screening is of better value than screening all patients. In many of the studies cited, age >40 years, being male and living in an urban area were significantly associated with a higher risk of DM.^13–15^,^20^,^23^ In India especially, other factors, such as smoking, past history of TB, increased waist circumference and pulmonary disease, were also associated with high rates of DM.^13–15^,^25–27^ As always, decisions about whom to screen for DM will depend on human resources, the technology available for DM testing and the feasibility of referral to DM clinics for confirmation of diagnosis and care.

When and how to screen TB patients for DM are two important programmatic issues that are yet to be fully resolved (Table 3). Although it is logistically easier to screen patients at the time of registration, and this has obvious advantages such as the potential to identify and control DM at the start of anti-tuberculosis treatment, previous studies have shown that TB as a chronic infectious disease may elevate blood glucose or glycosylated haemoglobin (HbA_1c_) levels, resulting in false-positive diagnoses.^1^,^28^ All DM diagnoses made at this early stage of anti-tuberculosis treatment should therefore be subject to later confirmation so that the patient is not erroneously labelled as having a life-long non-communicable disease (NCD). It is not yet known whether transitory elevated blood glucose levels in a TB patient are a marker for late DM, and it is advisable to recommend future follow-up DM testing in such patients. The most appropriate testing method for DM in the routine setting is also not resolved. In two large studies in India and China,^12^,^17^ TB patients were screened at the time of registration by asking first about the presence or absence of known DM, and in those denying any known disease by using random blood glucose measurements to identify those at risk, followed by fasting blood glucose (FBG) measurements in those needing to be further screened. This method identified those already with DM, who could be referred back to care for better control of their blood glucose and those with previously unrecognised new disease who could benefit from earlier diagnosis and treatment. However, FBG testing has low sensitivity. In India, HbA_1c_ performed better as a screening tool than FBG,^29^ and in a large DM prevalence study in China, screening with FBG missed nearly half of the DM patients diagnosed with a 2-h 75 g oral glucose tolerance test.^30^ The latter test, however, is cumbersome and inappropriate for screening individuals within routine general health services.

**Table 3.**
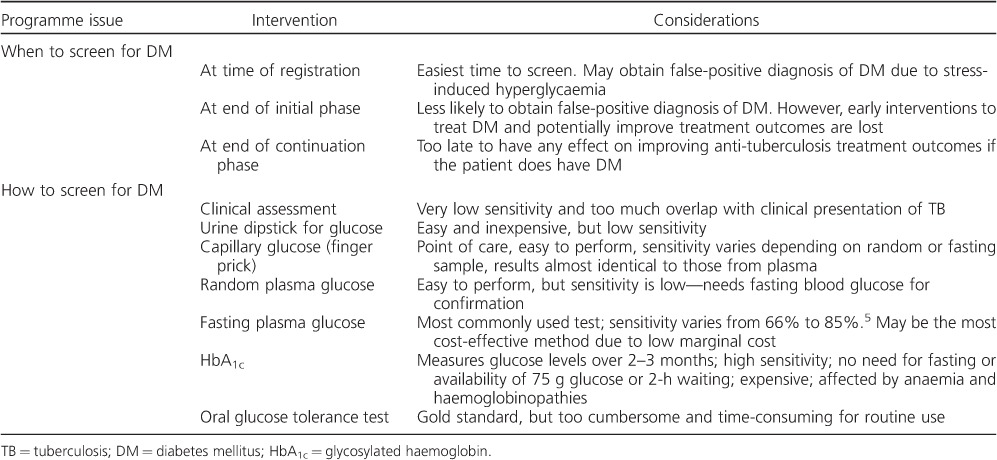
Programmatic issues related to the screening of TB patients for DM

In summary, HbA_1c_ has to be the gold standard measurement that programmes aim for, as this assesses blood glucose levels over a period of 2–3 months rather than on a particular day. Multiple efforts are now underway to produce low-cost, reliable assays for HbA_1c._ Other point-of-care glucose measurement technologies are being developed, and all of these should improve diagnostic screening in the future.^31^

## SCREENING PERSONS WITH DIABETES MELLITUS FOR TUBERCULOSIS

People with DM should be considered for systematic TB screening only in countries with a TB prevalence of over 100 per 100 000 population, as the number needed to screen to detect a new case of TB can be very high when TB prevalence is low.^10^,^32^ How best and how frequently this should be done at the programme level still requires further evaluation. In India and China, DM patients were screened for TB using a traditional symptom screen every time the patient visited the clinic, and those with positive symptoms were referred to TB services for investigation, primarily using sputum smear microscopy.^33^,^34^ This approach resulted in high TB detection rates that varied from 300 to 800/100 000 persons screened per quarter in China to 600–950/100 000 persons screened/quarter in India. However, a large proportion of these TB cases were already diagnosed and on treatment prior to screening; the cost-effectiveness of this approach thus needs further detailed evaluation.

There were several other operational and programmatic challenges, including 1) reluctance of busy DM doctors to take on the additional work needed to screen for TB, 2) the low sensitivity of current pulmonary TB diagnostic approaches that rely on sputum smear examination and chest radiography, and 3) difficulties in diagnosing extra-pulmonary TB. Further work is needed to determine whether screening using chest radiography, followed by rapid nucleic acid amplification technology for diagnosis, such as Xpert^®^ MTB/RIF (Cepheid, Sunnyvale, CA, USA), is feasible, more sensitive and cost-effective. Benefits of uniform or targeted screening should also be evaluated. In a large tertiary care hospital for DM in South India, important characteristics of DM patients that put them at higher risk of TB included older age, longer duration of DM, poor glycaemic control, higher frequency of alcohol consumption and lower body mass index; these characteristics could be used to determine who especially needs to be screened for TB.^35^

One simple, inexpensive, and as yet unevaluated method is to implement a major education programme for care givers and patients, so that persons with DM understand the risks of TB, recognise the symptoms and present to health care services when they think they might have TB. Such an approach might also help mitigate the risk of person-to-person TB transmission within DM clinics. There is currently no evidence to support screening for latent tuberculous infection (LTBI) in DM clinics, and this approach is not recommended in the WHO-Union Framework nor in the recent WHO guidelines on the management of LTBI.^10^,^36^

## TREATMENT AND CARE FOR PATIENTS WITH TUBERCULOSIS AND DIABETES MELLITUS

Most patients with dual disease are cared for by their respective programmes. However, an integrated, co-management approach might be a better option, and certainly from the patients' perspective. During the initial phase of anti-tuberculosis treatment, there is strict supervision and support that includes many encounters with health care staff. There are opportunities here for integrated health education and integrated clinical management. In the TB clinic, patients identified with DM could be referred to the DM clinic for diagnostic confirmation and advice about diet, exercise and drugs and then managed for DM back at the TB clinic during the course of anti-tuberculosis treatment. Once anti-tuberculosis treatment is completed, such patients should be referred permanently to the DM clinic, with vigilant follow-up to identify recurrent TB. A similar approach could be adopted for persons with DM who are diagnosed with TB, with the management of dual disease being centred at the TB clinic during the entire length of anti-tuberculosis treatment. This would require discussion, education, training and resources directed at the TB clinics, but as with HIV-associated TB this would be better for the patient, who would be regarded as one person with two diseases.

There are still uncertainties about the optimum treatment strategies in patients with dual disease; some of the key issues are highlighted in Table 4. Extended anti-tuberculosis treatment in DM patients is used in some places, and this has been the subject of some recent published research.^37^ However, the evidence for extending treatment beyond 6 months is weak, no randomised controlled trials have assessed whether extended or otherwise changed treatment regimens are more effective than standardised regimens, and the WHO does not recommend such a policy. It would seem sensible in those with dual disease to avoid sulphonylurea derivatives and treat DM with diet, lifestyle modifications, metformin and insulin instead, as these last two medications have little interaction with anti-tuberculosis drugs.^5^ There is also preliminary evidence to suggest that metformin may be an effective adjunct to anti-tuberculosis treatment by augmenting protective host-immune responses.^38^ Peripheral neuropathy from isoniazid can often be prevented by giving pyridoxine 12.5 mg daily, and this should always be administered in the presence of concurrent DM. Finally, little is known about facility-based TB transmission in DM clinics. In Mexico, 20% of DM patients with recurrent TB had re-infection with a different strain of Mycobacterium tuberculosis,^9^ and it is possible that recurrent disease may result from inadvertent exposure to undiagnosed TB in DM clinics.

**Table 4.**
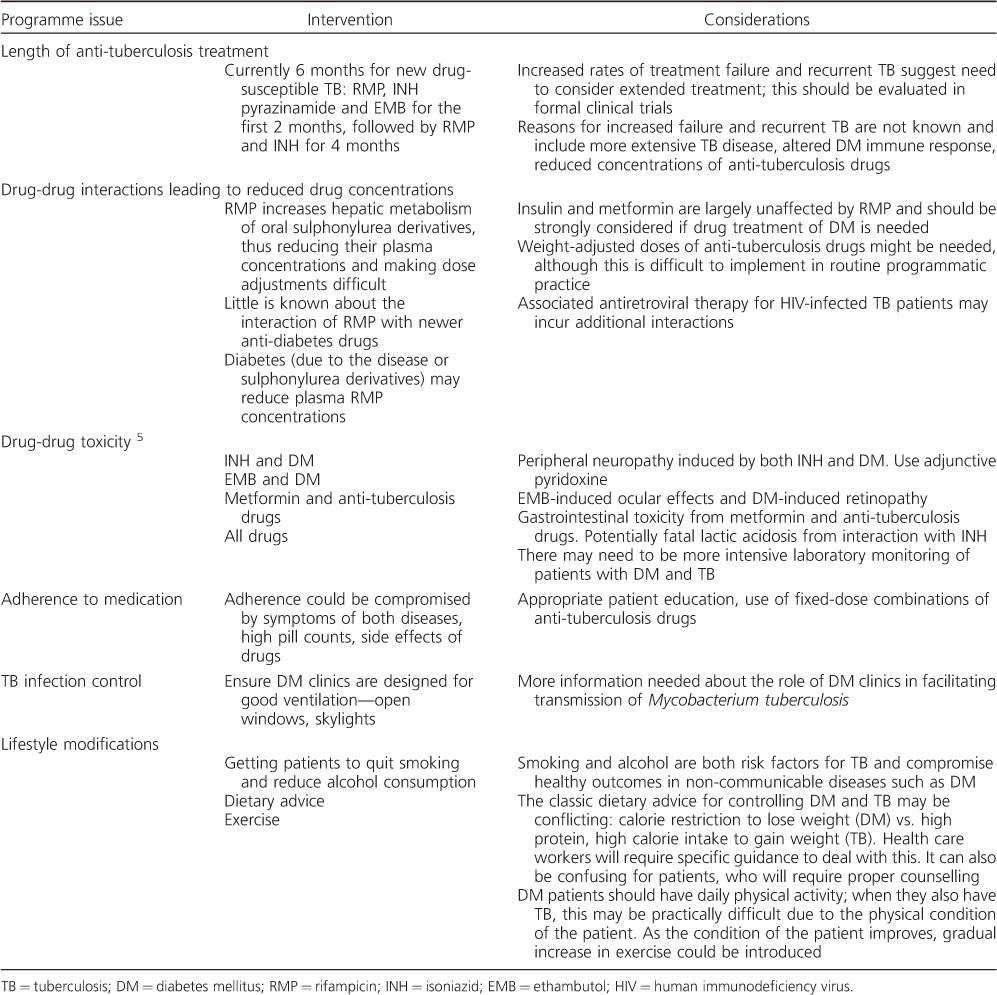
Programmatic issues related to the treatment and care of patients with both TB and DM

## MONITORING AND EVALUATION

**Figure 1.**
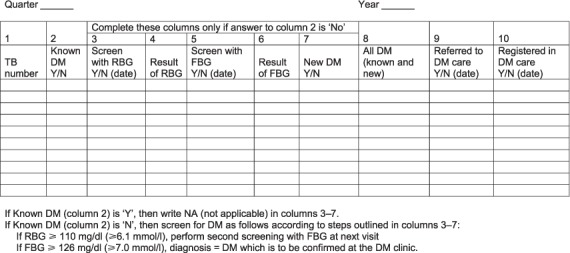
Page of a Tuberculosis-Diabetes Register showing how TB patients were screened for DM and the results recorded at TB Units, in India (adapted from 12). TB = tuberculosis; DM = diabetes mellitus; Y = yes; N = no; RBG = random blood glucose; FBG = fasting blood glucose.

**Figure 2.**
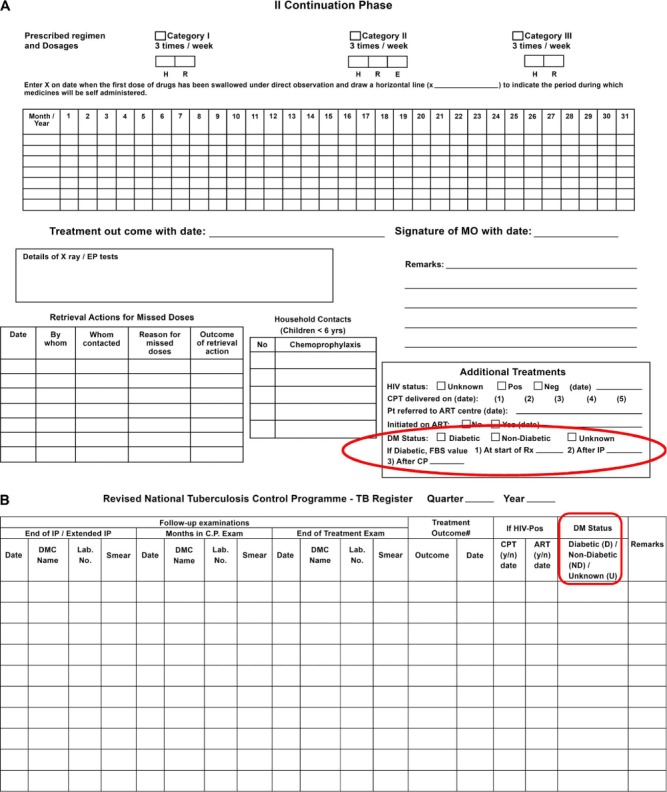
Recording of results of DM screening in **A)** the back of the TB treatment card and **B)** the right hand page of the patient TB register in India after the country had adopted a policy of screening all TB patients for DM. HIV= human immunodeficiency virus; DM= diabetes mellitus; DMC = designated microscopy centre; CPT = cotrimoxazole preventive therapy; ART = antiretroviral therapy; H = isoniazid; R = rifampicin; E = ethambutol; MO = Medical Officer; FBS = fasting blood sugar.

The cornerstone of good TB control programmes is a standardised monitoring and evaluation system providing quarterly reports on the number of patients registered for anti-tuberculosis treatment, the types and categories of TB and their treatment outcomes. It has thus been relatively easy to build into this system a monitoring and evaluation framework for DM screening, similar to what is currently being done for HIV/AIDS (acquired immune-deficiency syndrome) and antiretroviral therapy.^39^ In the large studies in India and China,^12^,^17^ TB patients were screened for DM according to a set algorithm, and results were recorded in a separate TB-DM register linked to the main TB patient register through TB registration numbers (Figure 1). The same format was used to prepare quarterly reports on aggregate data, provide an understanding of what steps worked and the results of each screening component. However, the quantity of data provided in such a monitoring and evaluation system is too much for a national TB programme that simply wants to know how many TB patients were screened, how many were diagnosed with DM, how many were referred for DM care and what the treatment outcomes were. Figure 2 (A and B) shows how this information was integrated into the TB treatment cards and TB patient registers in India after the country had made a policy decision in 2012 to screen all TB patients for DM.

**Figure 3.**
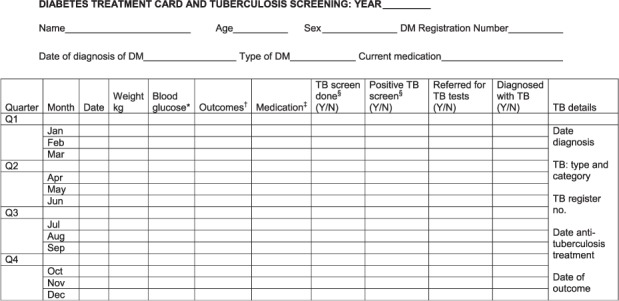
Treatment card used for screening persons with DM for active TB every time they attend DM clinics in India (adapted from^34^). *Fasting, random, post-prandial. ^†^Alive in care, died, lost to follow-up, transferred out. ^‡^Diet, oral medication, insulin. ^§^TB screen= ask about cough >2 weeks and/or suspicion of TB; positive TB screen= cough >2 weeks and/or suspicion of TB. TB= tuberculosis; DM = diabetes mellitus; Y = yes; N = no.

Recording the results of screening DM patients for TB has been a much more difficult exercise, largely due to the absence of any globally established cohort reporting systems for patients with chronic NCDs. In India and China, treatment cards for persons with DM were developed and used in clinics to record the outcomes of clinic visits, and in particular who had been screened for TB, who had positive symptoms suggestive of TB, who were referred for TB investigations and who were diagnosed with TB (Figure 3).^33^,^34^ This monitoring system captured the number of DM patients attending the clinic each quarter, but in many cases this included the same patients who had attended the clinic in previous quarters. However, because the clinics had not adopted any formal system for registering their patients, the cumulative number of patients ever registered, which increased each quarter as new patients were added to the pool, was not known. It was therefore not possible to obtain the patient denominators that are so crucial to calculate case detection rates. The answer lies in persuading NCD programmes to adopt a formal cohort analysis approach. This has been successfully used in hospitals in Malawi and primary health care clinics in the Near East,^40^,^41^ and deserves wider recognition.

## CONCLUSION

Given the accepted link between DM and TB and the escalating global burden of DM, which is set to exceed 500 million people by 2030, the inclusion of DM in strategic plans to control TB will become increasingly important in the next few years. More evidence is required to answer important questions about bi-directional screening in different settings, optimal treatment and care and integration of services, which could lead to better TB prevention, earlier diagnosis and start of treatment for DM and improved health outcomes for those with dual disease. The link between DM and TB and the framework for collaborative activities have the potential to stimulate and strengthen the implementation and scale-up of NCD care and prevention programmes. This may help not only to reduce the burden of non-communicable and communicable disease, it could also be a driver to strengthen health systems, a necessary pre-requisite for establishing universal health coverage. High-level political support within countries as well as international financial and technical support for disease programmes will be essential to move this agenda forward.
